# On the visual analytic intelligence of neural networks

**DOI:** 10.1038/s41467-023-41566-2

**Published:** 2023-09-25

**Authors:** Stanisław Woźniak, Hlynur Jónsson, Giovanni Cherubini, Angeliki Pantazi, Evangelos Eleftheriou

**Affiliations:** 1 IBM Research – Zurich, Säumerstrasse 4, 8803 Rüschlikon, Switzerland; 2 ETH Zürich, Rämistrasse 101, 8092 Zürich, Switzerland

**Keywords:** Computer science, Computational neuroscience

## Abstract

Visual oddity task was conceived to study universal ethnic-independent analytic intelligence of humans from a perspective of comprehension of spatial concepts. Advancements in artificial intelligence led to important breakthroughs, yet excelling at such abstract tasks remains challenging. Current approaches typically resort to non-biologically-plausible architectures with ever-growing models consuming substantially more energy than the brain. Motivated by the brain’s efficiency and reasoning capabilities, we present a biologically inspired system that receives inputs from synthetic eye movements – reminiscent of saccades, and processes them with neuronal units incorporating dynamics of neocortical neurons. We introduce a procedurally generated visual oddity dataset to train an architecture extending conventional relational networks and our proposed system. We demonstrate that both approaches are capable of abstract problem-solving at high accuracy, and we uncover that both share the same essential underlying mechanism of reasoning in seemingly unrelated aspects of their architectures. Finally, we show that the biologically inspired network achieves superior accuracy, learns faster and requires fewer parameters than the conventional network.

## Abstract (web summary)

Visual oddity tasks delve into the visual analytic intelligence of humans, which remained challenging for artificial neural networks. The authors propose here a model with biologically inspired neural dynamics and synthetic saccadic eye movements with improved efficiency and accuracy in solving the visual oddity tasks.

## Introduction

A long-term goal of artificial intelligence (AI) research is to devise cognitive systems that exhibit the capability of abstract reasoning, thus far deemed a distinctive characteristic of human intelligence. Not only is this a quest to improve our understanding of the nature of intelligence, but also may enable AI to assist humans in solving abstract complex tasks of repetitive nature or scale beyond the grasp of humans. In recent years, as deep learning has become more prominent, machine learning models have outperformed humans in several fields, including image classification and playing strategic games^1,2^, albeit relying on unsustainably increasing model sizes^3^. The energy requirements of these models are in stark contrast to ≈20 W required by the human brain^4^, whose principles of operation may provide crucial inspiration for improvements in AI, reducing the environmental footprint of existing solutions and enabling new low-power applications at the edge. Moreover, taking inspiration from brain’s operation during versatile tasks, such as those where abstract or relational reasoning capabilities are required, will pave the way for the development of novel valuable AI architectures. The interest in demonstrating applicability of artificial neural networks for reasoning has recently grown as more demanding tasks are being considered and large datasets are being created^5–9^

In an attempt to tackle these tasks, several neural network architectures have been proposed. A network architecture called Relation Network (RN) was proposed in ref. ^10^ to achieve state-of-the-art results on the CLEVR dataset^6^, a visual reasoning task with textual questions. In ref. ^10^ it was argued that RNs have a structure suitable for relational reasoning, as they learn to infer an existing relation between objects. The objects, or vector embeddings, are produced by a convolutional neural network (CNN) from an image. At this stage, RN shares the input processing part with Siamese networks^11^ and related similarity analysis architectures^12^, which evaluate the embeddings for similarity between a particular designated template or search image and other images, or even subparts of a larger image^13,14^. However, the subsequent evaluation of embeddings in RNs involves a specific comparison of embeddings for all pairs of objects combined with an embedding of the question in the task that enables to reason about relations between groups of objects. The initial model was tailored towards a visual question answering task with a single image, but an RN-based architecture appears to be useful across all kinds of reasoning tasks. A network called Wild Relation Network (WReN) was proposed in ref. ^5^ to solve Raven’s Progressive Matrices (RPMs), which involve selecting the correct pattern from eight candidate responses to complete a matrix with eight context patterns. This task is difficult for powerful deep networks, including CNNs and recurrent networks^5^. WReN surpasses their accuracy by applying RN modules to infer relations between the elements of matrices. For the solution of problems that require multiple steps of relational reasoning, for example Sudoku riddles, an advanced recurrent RN module has been developed in ref. ^15^. A theoretical framework to characterize what tasks a neural network can reason about was developed in ref. ^16^. Building on the observation that reasoning processes resemble algorithms, it explains the reasoning capabilities through comparisons of network’s computations to reasoning algorithms.

Another important class of tasks requiring analytic intelligence is the so-called visual oddity, first introduced in ref. ^17^ as part of a neuroscientific experiment to study the knowledge of core conceptual principles of geometry. This specific visual oddity task is defined on geometrical objects and consists of 45 distinct riddles designed to discover which basic concepts of geometry such as points, lines, parallelism, and symmetry, are understood by the participants of the study. Each riddle contains six frames, five of which include a geometrical concept being tested. One of the frames violates the geometrical concept and is called the oddity. The goal is to classify which one of the six frames is the oddity. The task was originally presented to two different groups of people: Americans and Munduruku, an Amazonian indigenous group. Individual riddle accuracies of both groups were recorded for children and adults. The results showed no significant difference in accuracies between the American and the Munduruku children. This suggests that the core knowledge of geometry required for solving the abstract problem of the visual oddity tasks is accessible to children also in the absence of schooling and experience with graphic symbols and geometrical terms. Simultaneously, while the results of Munduruku adults remained indistinguishable from these of Munduruku children, educated American adults scored significantly higher. The participants from the Munduruku population solved on average 66.8% riddles, performing well with the core concepts of topology, Euclidean geometry, and basic geometrical figures. Recognizing the oddity within geometrical transformations turned out to be significantly more challenging, thus suggesting that such transformations may represent inherently more difficult concepts, that through process of education become accessible to American adults solving ≈84.8% riddles. These results render the visual oddity task as an appealing abstract reasoning task to assess the accessibility of the visual abstract concepts in AI systems.

An approach to study the visual oddity task by resorting to a computational model was proposed in refs. ^18,19^, where the authors used the frames from the original work^17^ to first generate representations based on glyphs, while separately considering properties of edges, shapes, lines, points, etc. The model then adopts a structure-mapping engine to find the commonality across the frames. This is obtained by relying on analogical generalization to build up a representation of the common features in the images of a riddle. Individual images are compared to the generalization, and the odd image is singled out as the one that exhibits the lowest similarity. The model operates based on a predefined set of geometric concepts, grounded in psychological research, that are then used for qualitative comparisons of cultural differences in solving the riddles^19^.

Still, current models applied for visual analytic tasks operate markedly different from how the brain operates. Firstly, saccadic movements made by the human eye during inspection of different images play an important role while solving analytic tasks^20^. In the context of artificial neural networks, saccades were studied with two distinct motivations: predicting human saccadic movements^21,22^, and exploiting saccadic input, primarily in terms of reducing the amount of input data^23^; yet not from a perspective of their computational properties. Furthermore, signals from the eyes are processed by biological neurons, which can be modeled as spiking neural networks (SNNs) that represent a very efficient class of biologically inspired neural networks^24–27^ They are passing information through sequences of spikes, realizing an efficient sparse communication with all-or-none events. Concurrently, their rich internal dynamics modeling the temporal integration of incoming spikes from the synapses at the dendrites makes them well suited to tackling problems that involve the temporal evolution of the input information fed into the network^28^.

In this work, we take a biologically inspired perspective on investigating AI systems, which can be capable of solving analytic intelligence tasks. Specifically, we take substantial inspiration from biology in several aspects, including neural dynamics, neural architecture, and input image processing to improve the efficiency and to devise a novel architecture with reasoning capabilities. Initially, to provide an RN-based baseline, we develop an Oddity Relation Network (OReN) with a task-specific architecture suitable for the solution of the visual oddity task. Then, inspired by biology, we develop a new approach for solving analytic intelligence tasks. To that end, we focus on an implementation with SNNs leveraging their energy efficiency and rich temporal neural dynamics. We demonstrate that SNNs are also inherently capable of relational reasoning when coupled with a biologically realistic approach to input image processing. In particular, we investigate a novel perspective on the role of saccades as a computational primitive. We explore how they can constitute an important function in performing relational reasoning by delimiting the analyzed image from the context stored within the temporal neural dynamics. Our proposed approach demonstrates that relational reasoning becomes possible in a biologically inspired architecture through interactions of neural dynamics and saccadic inputs. Furthermore, when compared with the conventional deep learning approach of OReN, it provides superior accuracy and smaller model sizes for solving the visual oddity task. It turns out that, although the accuracy of artificial neural networks extensively trained in a machine learning setup cannot be directly comparable to that of humans, both OReN and SNNs solve on average proportionally more riddles than humans.

## Results

### Generation of visual oddity riddles

**Fig. 1:**
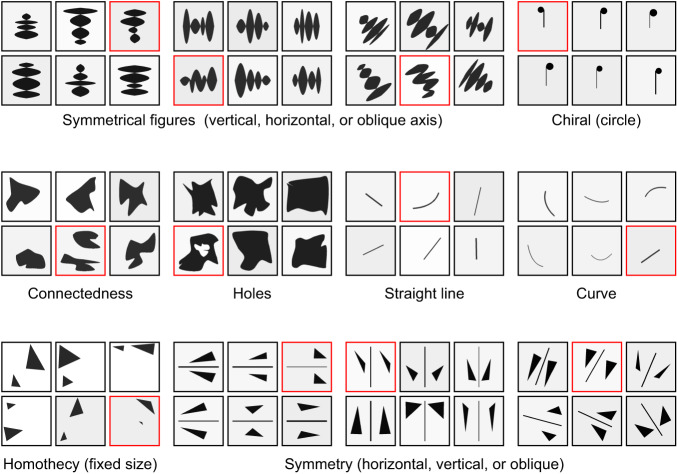
Generated visual oddity riddles. A set of twelve visual oddity riddles representing different core concepts with odd images marked with red borders, analogous to the original neuroscientific experimental work^17^.

Following the deep learning paradigm, it is essential to use large training datasets to achieve high performance. For instance, to train models solving RPMs, a large dataset comprising so-called procedurally generated matrices was developed^5^. The generation procedure consists of creating an abstract structure for each matrix by first sampling triples from three primitive sets (relation types, object types, and attribute types) that represent the challenge posed by the matrix. For example, such a triple could consist of progression, shape, and number. From the sampled triple, attributes are also sampled from the primitive sets for each type in the triple. However, determining a similar structure for the visual oddity task is infeasible, because of the variability of the frames within a single riddle and across the 45 different riddles. To that end, we created a dataset where different samples of each riddle with a specific underlying geometrical concept are procedurally generated by 45 riddle-specific generators. Twelve samples of generated riddles representing different core concepts are illustrated in Fig. 1. For each riddle, a key spatial concept is present in five of the six images. All other spatial features are irrelevant and may vary randomly. For example, in the top left sample in Fig. 1, five out of six images follow the key concept of vertical symmetry, whereas the exact size and form of each shape varies. The details of the generation procedure are explained in “Methods” and in Supplementary Note 1, with code link available. Training and test examples for all riddles are provided in Supplementary Fig. 1.

As mentioned earlier, the visual oddity task evaluates the capability of making analogies by relying on geometric concepts such as points, lines, and angles. Therefore, the detection of similarities is key to solving the riddles. For humans, abstract reasoning is crucial to find a correct answer, as no two images are the same and there are several ways to detect an odd image. In fact, to succeed, the participants must infer the geometrical relationship between the array of images. Therefore, the challenge for neural networks lies in whether they can infer the abstract geometrical relationships between the images. Training different neural network architectures to solve the visual oddity task provides an insight into their relation-forming capabilities.

### Oddity Relation Network (OReN)

RNs provide a general solution to relational reasoning in neural networks that extract information about a relation that may be formed by all pairs of objects in certain contexts. They exhibit three main characteristics, which make them well suited for solving relational reasoning tasks. First, these networks are capable of learning to infer the existence of object relations, as all pairs of objects and their potential relations are considered. Second, they compute each relation by a single function, typically a multi-layer perceptron (MLP), which leads to high data efficiency. The MLP operates on a batch of object pairs, where each sample of the batch is drawn from the same set. Third, they operate on a set of objects for which a specific order is not required, thus ensuring that the RN’s output contains information that reliably represents the relations within the set.

As mentioned in the introduction, the WReN is an RN that was specifically proposed to solve RPMs. Consequently, it is particularly appropriate to address reasoning tasks that require the detection of similarity between images. The model computes pair-wise relations between context panels and response panels in an RPM dataset to infer the relations between the eight matrix elements and the eight candidate answers. The information pertaining to context-context relations and context-multiple-choice relations is then integrated to provide a score for the selection of the answer. For the visual oddity task, there are neither context panels nor choice panels, as all frames can be classified as the oddity. Therefore, we modify the WReN to obtain the OReN and investigate its capability to solve all 45 riddles of the visual oddity task. In the OReN, first the pair-wise relations between each panel and the remaining five panels are formed, then the resulting information is integrated to obtain a score for the identification of the oddity.

In contrast to the computational model in ref. ^19^, we put an emphasis on working directly with the raw image data and have the network discover the relevant concepts without prior information through the process of training. For the experiments, we use datasets generated as described in “Methods”. Each sample in a dataset comprises six frames and a label, which indicates the index of the oddity. Each frame is first input into a 5-layer CNN, referred to as the vision model. For every frame *k, k* ∈ [1, 2,…, 6], the vision model outputs a frame embedding **η**_*k*_, represented as a vector with *D* dimensions. For each frame embedding **η**_*k*_, pairs are generated by ordered concatenation with all six frame embeddings. A total of 36 pairs are thus generated. Each pair is input to a function, *g*_*θ*_, parameterized by a neural network *θ*. The six outputs of *g*_*θ*_ corresponding to frame *k* are summed up for each frame. The summed output of each frame is input to a second function, *f*_*φ*_, also parameterized by a neural network *φ*, to calculate the final score. The score for each frame *k* is then calculated as

1 $${q}_{k}={f}_{\varphi }\left(\mathop{\sum }\limits_{i=1}^{6}{g}_{\theta }\left({{{{{{\boldsymbol{\eta }}}}}}}_{k},\,{{{{{{\boldsymbol{\eta }}}}}}}_{i}\right)\right).$$

**Fig. 2:**
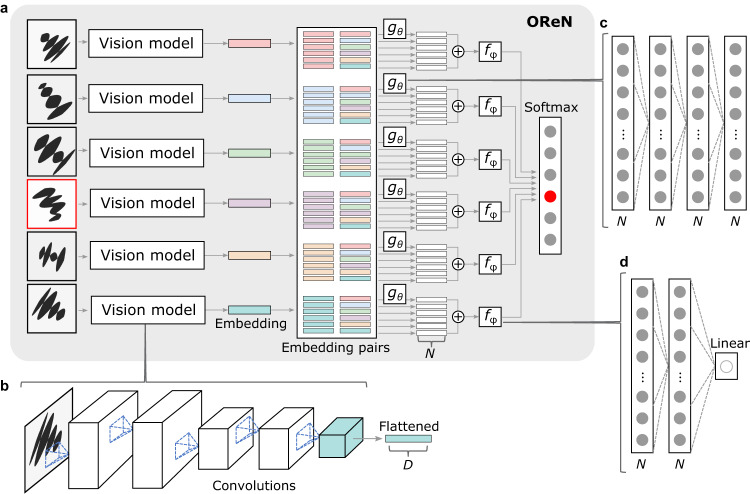
OReN architecture. **a** Generic architecture showing the vision model, the formation of embeddings and embedding pairs, as well the relation and decision stages. **b** Realization of the vision model by a CNN producing *D*-dimensional embeddings. **c** Realization of the function *g*_*θ*_ through a four-layer MLP with *N* rectified linear neurons per layer. **d** Realization of the function *f*_*φ*_ with a three-layer MLP with two *N*-sized rectified linear layers and a single linear output neuron yielding the score.

A softmax function is finally applied across all scores to determine the probability of each frame being the oddity. A generic architecture for the OReN is illustrated in Fig. 2, with details described in “Methods”. This figure also depicts the generation of the vector embeddings from the images of a riddle by the vision model.

### Saccadic neural network

The human approach to analytic reasoning differs from the operation of relational networks, such as OReN, starting with the manner the input stimuli are delivered from the eyes. Solving analytic reasoning tasks, where the inspection of various images is required, involves a series of repeated back-and-forth rapid eye movements, called saccades. The saccades allow the brain to compare all candidate images over a certain period and reach a conclusion, as observed in human subjects solving the RPM task^20^. Moreover, the operation of the biological neurons in the brain is characterized by spike-based communication and rich temporal neural dynamics that is often abstracted into a so-called leaky integrate and fire (LIF) neuron model, used widely in SNNs^25,29^, Therefore, it is appealing to explore analytic reasoning for the visual oddity task taking direct inspiration from a more biologically motivated approach.

**Fig. 3:**
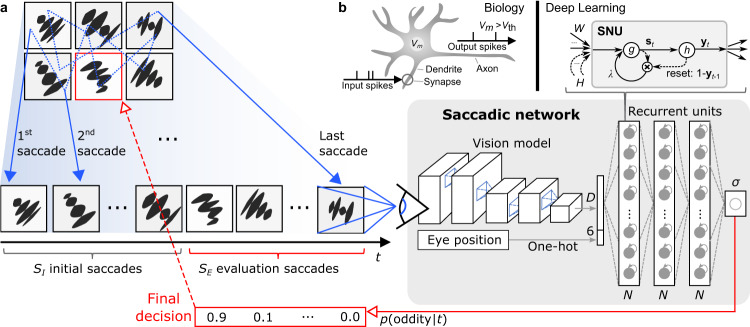
Visual analytic reasoning approach with a saccadic network. **a** The recurrent units receive *D*-dimensional image embeddings and eye-position inputs from a series of simulated saccadic eye movements and perform reasoning using the internal temporal dynamics. After *S*_*I*_ initial saccades, the final decision is determined from the outputs of the final sigmoidal (*σ*) neuron over *S*_*E*_ evaluation saccades. **b** The neural dynamics of biological neurons are incorporated into a deep network through SNUs, where each SNU represents an abstraction of the LIF model that integrates inputs from synapses into its membrane potential *V*_*m*_, and generates an output spike each time *V*_*m*_ crosses the spiking threshold *V*_th_.

To this end, we synthesize a series of eye saccades over the candidate frames and input them into a temporal saccadic neural network model, as illustrated in Fig. 3a. To ensure a balanced distribution of the vision input stimuli, i.e., that each candidate frame is observed the same number of times, we construct the input as a series of six random permutations of all six candidate frames, resulting in 36 time steps simulating a series of saccades. At each time step *t*, the saccadic network outputs its belief that the currently presented frame is the oddity: *p*(oddity|*t*). During testing, the initial *S*_*I*_ = 18 saccades are used to initialize the temporal dynamics of the reasoning neurons, whereas the probabilities obtained during the *S*_*E*_ = 18 evaluation saccades are integrated in order to provide the final decision.

The architecture of the saccadic network model is biologically inspired in several aspects. The vision model is a CNN that is reminiscent of the receptive fields in the visual cortex^30^. For a fair comparison with OReN, the same CNN structure is used in the saccadic network. Then, the recurrent neural units implement the temporal dynamics found in neocortical neurons. Specifically, as illustrated in Fig. 3b, the LIF abstraction of the biological neurons is simulated using a deep learning recurrent unit following the SNU approach^31^. The state equations for a layer of SNUs are

2 $${{{{{{\bf{s}}}}}}}_{t}	=\, g\left(W{{{{{{\bf{x}}}}}}}_{t}+H{{{{{{\bf{y}}}}}}}_{t-1}+{{{{{\rm{\lambda }}}}}}\odot {{{{{{\bf{s}}}}}}}_{t-1}\odot \left(1-{{{{{{\bf{y}}}}}}}_{t-1}\right)\right)\\ {{{{{{\bf{y}}}}}}}_{t}	=\, h\left({{{{{{\bf{s}}}}}}}_{t}+{{{{{\bf{b}}}}}}\right),$$

where **x**_*t*_ denotes the input vector at time *t*, **s**_*t*_ indicates the internal state vector modeling the membrane potentials *V*_*m*_ for each neuron, **y**_*t*_ denotes the output vector, and $g$ and *h* represent the input and output activation functions, respectively. Furthermore, *W* and *H* represent input and recurrent synaptic weight matrices, *λ* denotes a leak parameter, **b** is a vector of biases that determine the firing thresholds, and $\odot$ denotes point-wise multiplication. For the saccadic network, we employ three SNU layers, with *N* units each, followed by a single sigmoid readout neuron, that provides the belief value at its output. This formulation, which is relying on a deep learning approach, enables to operate in the spike-based SNN mode with SNUs assuming *h* = $\varTheta$, the Heaviside function, as well as in the standard artificial neural network mode with soft SNUs assuming *h* = $\sigma$, the sigmoid function, or even to use the popular LSTM units. This allows us also to compare the performance of the biologically inspired neural units with that of common deep learning recurrent units.

The key feature of the stateful neurons considered here consists in the processing of the current input within the context of the neuronal state that reflects the information from the past inputs. In other words, both the comparison of embeddings and the accumulation of information is performed over time, rather than over space as is done in RNs. Importantly, we show that the obtained membrane potential integration, in conjunction with a biologically realistic saccadic input stream, enables analytic reasoning. Common stateless deep networks struggle to solve analytic reasoning tasks. Our initial attempts to solve the visual oddity task with fully connected networks or even ResNet architectures led to ≈17% accuracy, which corresponds to guessing by chance. The essential functionalities introduced in the RN architecture, which enable analytic reasoning in such networks, are the addition of the processed representations and the reuse of the *g*_*θ*_ and *f*_*φ*_ modules for all the frames. Apparently, the first functionality is inherently present in neural recurrent units, such as SNUs, where an input **x**_*t*,_ transformed by the weights *W* is added to the previously processed inputs represented in the state **s**_*t*−1_. Furthermore, the idea of RN to process a series of inputs by replicating the same parameters in space, see Fig. 2, is naturally realized by a series of saccadic movements, where each input is processed by the same units, thus reusing the same synaptic weights *W*. The two biologically realistic aspects, namely the stateful operation in combination with a saccadic input sequence, provide the means for analytic reasoning.

### Can biologically inspired neural networks compete with relation networks?

We compared the accuracy of OReN and saccadic networks in two setups. First, in a separate training setup, in which networks were trained and evaluated for each riddle separately. Second, in a joint training setup, in which a single network was trained and evaluated on all riddles. In both setups, we varied the layer size *N* following a geometric progression, from 16 to 256 and from 64 to 4096, respectively, to study the dependency between the model size and accuracy for each architecture and unit. For a saccadic network we considered four types of units in the reasoning part: SNU-based spiking LIF neurons (SNN), soft SNUs (sSNU), soft SNUs with layer-wise recurrency (sSNU-R), and standard LSTMs. Details are described in “Methods”.

**Fig. 4:**
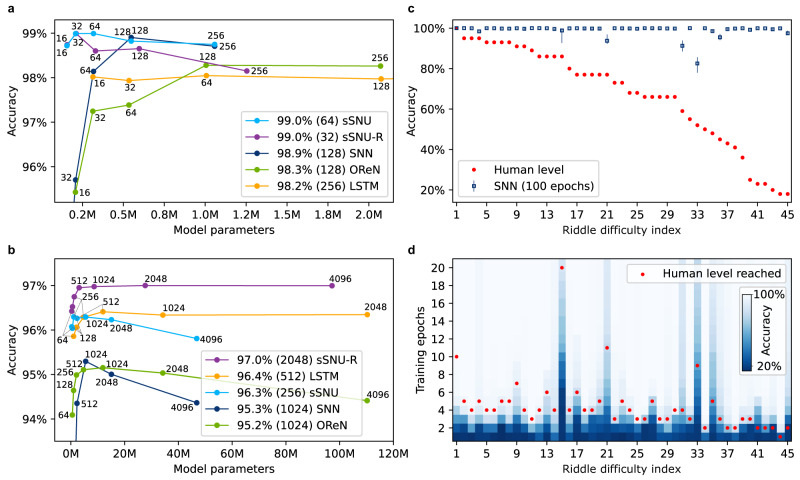
Accuracy comparison. **a**, **b** Average test accuracy for different model sizes (*N* value reported next to each point) for separate (**a**) and joint (**b**) training setup. Legend labels report the best average accuracy and the corresponding *N* value for each series. See Supplementary Note 3 for detailed values. **c** SNN (*N* = 128) separate training accuracy with min/max error bars plotted along with the human-level accuracy, for riddles ranked by difficulty level for humans reported in ref. ^17^, for which a difficulty index ranging from 1 (the easiest) to 45 (the most difficult) is assigned. **d** Test accuracy evolution over the first 20 training epochs with marked epochs when reaching human-level accuracy obtained in a different setup, described in text.

The results for the separate setup are presented in Fig. 4a. OReNs achieved an impressive average accuracy of 98.3% for *N* = 128 (≈1.0 M model parameters). This demonstrates that the RN-based approach enables the visual oddity task to be effectively solved. Saccadic networks with LSTM units performed slightly worse with 98.2%, while requiring many more parameters, ≈4.7 M for *N* = 256, for legibility reasons not shown within the plot boundary—see Supplementary Note 3 for a table with comprehensive results. Remarkably, the saccadic networks operating with much simpler biologically inspired dynamics achieved the best result of ≈99% using the fewest parameters: ≈0.5 M (*N* = 128) for SNN, ≈0.3 M (*N* = 64) for sSNU and ≈0.2 M (*N* = 32) for sSNU-R. Even the 98.7% accuracy of the smallest *N* = 16 sSNU saccadic network with ≈0.15 M parameters surpassed both the highest result of 98.2% (≈4.7 M) of the saccadic architecture with LSTM units, commonly used in machine learning, as well as of 98.3% (≈1 M) of OReN architecture. These facts illustrate potential benefits of incorporating more biological inspiration into AI architectures that can provide a more favorable parameters vs. accuracy characteristics and reduce the model sizes by an order of magnitude.

Figure 4b illustrates the results for the joint training setup, which is more challenging as the network needs to capture all visual concepts and seamlessly switch between recognizing 45 different kinds of oddities. In consequence, larger models were required to learn well, and the final accuracy levels dropped by ≈2%. OReN in the best performing configuration of *N* = 1024 (≈12 M) obtained 95.2%, which is a very good result in absolute terms. However, all saccadic networks performed better, reaching a top accuracy of 97.0% for sSNU-R with *N* = 2048 (≈28 M). SNN and sSNU achieved 95.3% with *N* = 1024 (≈5.5 M) and 96.3% with *N* = 256 (≈1 M), respectively. In comparison with the soft versions of SNU, the accuracy of the spiking version decreased relatively more, whereas the saccadic network with LSTM units performed quite well, achieving 96.4% for *N* = 512 (≈12 M). Overall, considering the number of parameters vs. accuracy characteristics, biologically inspired sSNU-R and sSNU remained the most favorable options also in the joint case, achieving high accuracy using ≈1.3 M (*N* = 256) and ≈1 M (*N* = 256) parameters, respectively. Comparing for instance 96.8% accuracy of sSNU-R ≈1.3 M (*N* = 256) with the best performing OReN reaching 95.2% and LSTM-based saccadic network reaching 96.4%, where both have ≈12 M parameters, the networks with biologically inspired neural dynamics demonstrate substantial advantages.

The average accuracy achieved by OReNs and saccadic networks is very high, yet some riddles are consistently more challenging than others. The prior art approach utilizing the computational model explored how the presence of particular concepts makes a riddle difficult for humans. That model’s accuracy was found to be significantly correlated with human performance^18,19^, As the concepts present in our generated dataset directly correspond to the ones from the original experiment, we perform a similar comparison based on the detailed accuracies reported for the Munduruku participants^17^. Figure 4c depicts the human performance for the riddles ranked by increasing difficulty level for humans along with the accuracy for the most biologically plausible of our models: a saccadic network with spiking neurons (*N* = 128). Although for some more difficult riddles, e.g., 31 or 33, the final accuracy indeed dropped, we observed no statistically significant correlation of the final accuracy-dependence on the riddle difficulty index. This consistently high accuracy, surpassing 66.8% accuracy of Munduruku and ≈84.8% accuracy of adult Americans^17^ as well as 86.7% of the computational model^19^, stems from a markedly different setup from the original visual oddity experiment.

It is important to understand that although our accuracy values quantitatively measure network’s accuracy on riddles analogous to the original experiment, qualitatively they are not directly comparable with human accuracy. Firstly, humans solve the riddles through generalization of analytic reasoning principles acquired in various contexts throughout their lives, whereas in our setup the networks were trained to discover these principles from examples directly reflecting the riddles. Following the standard machine learning approach, we obtained the training, validation, and test datasets by splitting multiple instances of each riddle generated from the same distribution. Secondly, the number of examples presented during training and testing of our models is substantially higher than just a single presentation in the original experiment. On the one hand, a high number of testing examples provides a more statistically accurate performance measure of our models than the original experiment with one example per riddle presented to each participant. On the other hand, a high number of training examples provides a substantial advantage to our models. Figure 4d illustrates how many training epochs were required to reach the human-level accuracy by the SNN saccadic model. On average it required ≈3.5 epochs, corresponding to presentation of 8932 examples. For comparison, the best OReN model with *N* = 128 required ≈4.1 epochs, corresponding to 10,524 examples. Devising machine learning systems operating in a more human-like manner and in human-comparable setups remains an important research question. In this context, human performance remains quite impressive.

**Fig. 5:**
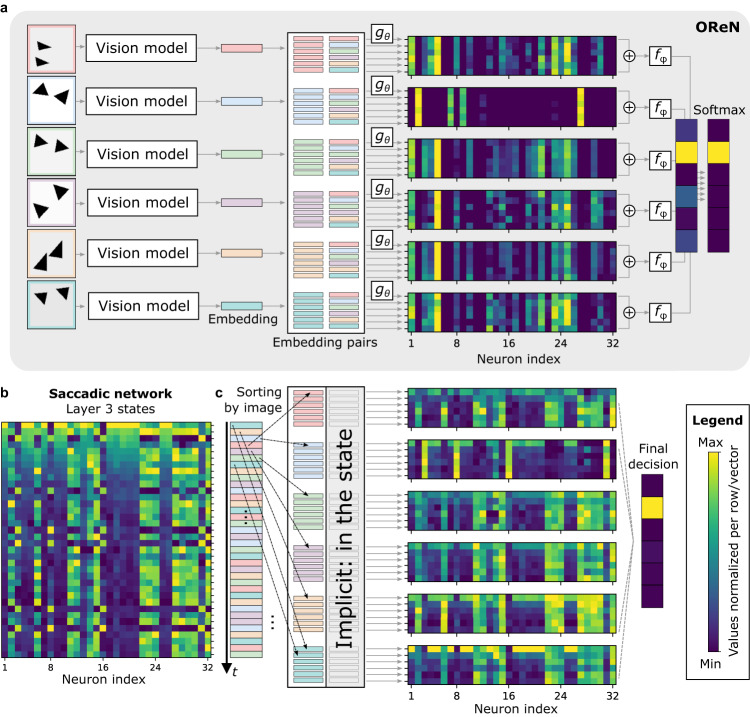
Activity comparison of an OReN and a saccadic network using spiking neurons. *N* = 32. **a** OReN identifies the oddity through calculation of *g*_*θ*_ for pairs of input embeddings, followed by aggregation and scoring through *f*_*φ*_. The embedding pairs comprising the oddity as the first element lead to a visually distinctive *g*_*θ*_ activation map for the oddity. **b** The saccadic network receives a temporal stream of inputs that lead to the evolution of the internal states, *V*_*m*_, visualized for Layer 3. **c** Membrane potentials sorted by the order of the first OReN embedding pair item exhibit similar visual distinction for the oddity. Visualized values were normalized row-wise, i.e., over 32 neurons.

Although OReNs and saccadic networks seemingly operate based on different principles, the discussion in the previous sections unveiled similarities of the reasoning mechanisms between the two approaches. We conjectured that OReN’s reasoning mechanism relies on a series of comparisons in space, whereas the saccadic network performs them over time. We validate these statements by visualizing the internal representation during inference of trained OReNs and saccadic networks with SNNs, with *N* = 32 for both. The activity maps calculated in an OReN through *g*_*θ*_ and *f*_*φ*_ are depicted in Fig. 5a. The second activity map is visually distinct from others, and after the application of the scoring function *f*_*φ*_, the OReN correctly identifies that the second input image is the oddity. Next, the same set of images is presented to the saccadic network through a series of saccades. The state values of Layer 3 are visualized in Fig. 5b. Gradual and abrupt decrease of *V*_*m*_ over time (the time axis is vertical) corresponds to membrane potential leakage with parameter *λ* and resetting after spikes, respectively. In this form, it is difficult to compare both models. However, as seen in Fig. 5c, when membrane potentials are sorted as if the input saccades would follow the order of the first items from the OReN embedding pairs, the membrane potentials of Layer 3 neurons become visually distinctive for the saccades comprising the oddity, similarly to the activity maps in the OReN. This functional correspondence between the *V*_*m*_ and the activity of OReN is qualitatively different than the commonly observed similarity between the ANN activities and the SNN spiking rates^32^. Eventually, both models correctly identify the second image as the oddity based on the same principle of comparisons. In the OReN the comparisons are performed in space, with each pair of input images explicitly presented at different inputs of the network at the same time. In the saccadic network, the temporal dynamics implicitly stores the context and it is possible to operate with only one input image observed at a time. This is similar to how the brain analyzes the sensory inputs from saccades over time and compares information to the past context to perform analytic reasoning.

We investigated the visual oddity task, which requires a high level of visual analytic intelligence, from the perspective of both RNs and biologically inspired saccadic architectures. We first generated a large dataset of visual oddity riddles that represent the core concepts to be tested and allow thorough network comparisons. Then we explored two avenues, one where the RN approach was extended to give origin to an OReN capable of tackling the visual oddity task, the other where a novel biologically inspired architecture processes a stream of the same input panels one-at-a-time to determine the oddity. Both avenues lead to networks that are characterized by the capability of establishing relations between the images of a riddle, and outperform previously proposed computational models, as well as previously reported average human performance, the latter being achieved though in a different setup and context of lifetime experience. Furthermore, we validated the conjecture that saccadic networks evaluate the image relations sequentially over time, whereas the RNs operate in parallel over space. Our findings indicate that saccadic network models with biologically inspired units achieve better performance than RNs and require a significantly lower number of model parameters, providing a potential avenue for improving the efficiency of deep learning and a prospective widespread applicability in edge applications. In particular, vision-related applications, such as anomaly detection or object classification at the edge may yield qualitative improvements through inclusion of abstract reasoning capabilities and our saccadic approach, tailored to more realistic human-like selective observation of the world.

## Methods

### Generation of riddles

To use standard supervised learning methods to solve the visual oddity task we need a dataset for each of the 45 different riddles. As the original paper^17^ only contains one sample per riddle, we created a dataset where each riddle is procedurally generated. Below we describe the riddle generation procedure. Each of the 45 different riddles has individual attributes. For the dataset generation process, we consider the attributes of each riddle in a procedural way. In order to ensure as much variability as possible in the dataset, we include all the possible attributes as variables that take randomized values. Each sample in the dataset is a combination of six 100 × 100 frames and a label, which indicates the index of the oddity in the array. Every frame we generate is an 8-bit grayscale figure. Each pixel has 256 possible values ranging from 0 (black) to 255 (white). Each frame has a background grayscale value ranging from 235 to 255. For each frame, we additionally randomly sample a grayscale value between 0 and 61 to be used for all surfaces, edges, and points. The generated frames are compared with each other to ensure that they are all pixel-wise unique. We generate 45 small datasets of size 3840 separately for each specific oddity type, and one large dataset of size 108,000 comprising all oddity types. Each dataset is split into training, validation and test set, with sample count following a ratio of 4:1:1, respectively.

When generating a dataset for a single riddle, the riddle index (ranging from 1 to 45) is fixed, so it will be the only riddle for which frames are generated. In contrast, when generating a dataset containing multiple riddles, the riddle index is sampled randomly from the set of riddles for each sample in the dataset. Each riddle has its own generator that first generates five frames that contain the common conceptual geometrical property of the riddle, called non-oddities. Afterward, the riddle generator generates one frame that does not contain the property, i.e., the oddity. The six frames are then randomly shuffled and the index of the oddity is returned as the label. All sizes are determined in a percentage of the maximum frame size to allow the generation of various frame sizes. An example of each generator output can be seen in Supplementary Fig. 1, where the oddity of each riddle is also highlighted.

All 45 riddles are divided into eight categories as defined in ref. ^17^, where each category corresponds to one of the considered geometrical challenges. The details of each category along with the variables used in each riddle are listed in Supplementary Note 1. Some variables are used for every riddle in a category and some are used for several categories, as indicated in Supplementary Tables 1–8.

### Detailed architecture of OReN

The OReN architecture, depicted in Fig. 2a, was implemented in TensorFlow 1. It comprises a CNN vision model that is applied six times to produce 3200-dimensional embeddings of the six candidate frames. The embeddings are paired into 6400-dimensional vectors that are processed by the MLP *g*. The resulting vectors are summed and scored by a second MLP *f*. The oddity is determined by detecting which neuron yields the highest value from the final softmax layer.

The CNN vision model, depicted in Fig. 2b, receives an input image rescaled to 80 × 80 and processes it using five 32-channel convolutional layers with 5 × 5 kernels and no border padding. Each convolution is followed by a batch normalization. The first, third, and the fifth convolution layers are followed by a dropout operation with 0.3 dropout rate. The second and the fourth convolution layers are followed by 2 × 2 max pooling with horizontal and vertical strides equal to two, and zero padding at the borders. The output of the final convolutional layer is flattened into a 3200-dimensional embedding vector. A detailed visualization of the vision model is included in Supplementary Note 2.

The *g* function is instantiated for a series of 6400-dimensional vectors comprising concatenated pairs of embeddings, as depicted in Fig. 2c. In each case, the vector is processed by a four-layer fully connected network with *N* rectified linear units per layer. Each layer is followed by a dropout operation with 0.3 dropout rate. The output is an *N*-dimensional vector. For each embedding pair group, six such vectors are added and form an input to the *f* function, as illustrated in detail in Fig. 2d. This input is processed by two fully connected layers with *N* rectified linear units per layer, followed by a single linear neuron that produces the final score for the considered group of the embedding pairs. The six scalar scores from the six instantiations of *f* are processed by a softmax layer that provides the final probability distribution, where the neuron yielding the highest value indicates the index of the oddity. A categorical cross-entropy loss between the output probabilities and the ground-truth indices of the oddities is minimized during training using the Adam optimizer.

### Detailed architecture of saccadic network

The saccadic network architecture, depicted in Fig. 3b, was implemented in TensorFlow 1 and comprises the same CNN vision model as the OReN network. For each saccade, the vision model computes a 3200-dimensional embedding that is concatenated with the current eye position encoded as a one-hot vector (all zeros except the position corresponding to the index of the currently examined panel). The concatenated vectors are processed by three consecutive fully-connected layers with *N* recurrent units.

The recurrent units are either LSTMs or SNUs. The default TensorFlow 1 configurations of activation functions, parameter initializers and other settings are used unless stated otherwise. The specific hyperparameters for SNUs are the input activation function $g$ that is set to identity function (no input activation) and leak parameter *λ* that is set to 0.8. SNUs in SNN configuration have the activation function *h* set to the step function and in sSNU configuration it is set to the sigmoid function. SNUs with letter R in suffix include recurrent connections matrix *H*, that is skipped otherwise. The biases are initialized to −1.0.

The outputs from the recurrent units are processed by a single stateless sigmoidal output neuron that provides the output value for the current saccade. A series of saccades leads to a sequence of sigmoidal outputs, for which the error is minimized using a binary cross-entropy loss and Adam optimizer. The binary cross-entropy loss is masked, so that it considers only the relevant saccades. The outputs for the first two saccades are masked, as the appearance of the third distinct frame is the earliest possible moment when it becomes possible to conjecture which frame is the oddity.

### Reporting summary

Further information on research design is available in the Nature Portfolio Reporting Summary linked to this article.

### Supplementary information

Supplementary Information

Reporting Summary

## Data Availability

Dataset generation details are described in “Methods” and Supplementary Note 1. The generated files are available from the corresponding author upon request due to their large size. Preferred approach is to generate the dataset using the published source code. Detailed raw values from the figures are included in Supplementary Note 3.

## Code availability

Visual oddity dataset generation code is available at https://github.com/IBM/visual-oddity.
