# Supplementary material for: On the visual analytic intelligence of neural networks
Source: Nat Commun. 2023 Sep 25;14:5978. doi: 10.1038/s41467-023-41566-2 (PMC10520053; doi:10.1038/s41467-023-41566-2)
Supplement: Supplementary file 1 — Supplementary Information [file 41467_2023_41566_MOESM1_ESM.pdf]

Supplementary Information

On the visual analytic intelligence of neural networks

Stanisław Woźniak[1]*, Hlynur Jónsson[1,2], Giovanni Cherubini[1],
Angeliki Pantazi[1], Evangelos Eleftheriou[1]

[1] IBM Research – Zurich; Säumerstrasse 4, 8803 Rüschlikon, Switzerland.
[2]ETH Zürich; Rämistrasse 101, 8092 Zürich, Switzerland.

*Corresponding author. Email: stw@zurich.ibm.com

Supplementary Note 1: Generation of riddles

The details of variables used for generation of the riddles are described below, starting with the variables common to several or all categories of the visual oddity riddles.

Common variables

Background Color: As stated earlier, every frame has a random background grayscale value between 235 and 255.

Color: The 8-bit grayscale value variable used for all surfaces, edges and points of the riddle. The variable is used for all riddles.

Height and width: Height and width are two separate variables used for rectangular shaped objects to randomly decide the size of the shape.

Center: Randomly generated x and y variables determining the coordinates of the center of an image on the x-axis and y-axis, respectively. Both variables are within a predefined frame boundary to make sure the image does not flow outside the frame.

Rotation: Randomly generated rotational value between 0 and 360 deg to rotate an image around its center.

Line length: Similar to rectangle height and width but for the length of a line.

Line width: Width of lines for riddles including lines. Minimum and maximum values are decided based on the frame size.

Point radius: Points in riddles are modeled as circles with a randomly sampled radius in proportion to the frame size.

Radius: Circle radius of riddles containing circles. The radius variable is also adopted for triangles and quadrilaterals by modeling a circle as the circumcircle of either shape.

Initial training

Variables used for the two riddles in the ”Initial training” category are shown in Supplementary Table 1. The grayscale value variable is used for filling the oddity shape in the Color riddle. The rotation variable in the *Orientation* riddle has a minimum rotation of 15 deg.

Riddle type	Height & width	Center	Rotation
Color	X	X	
Orientation	X	X	X

Supplementary Table 1: List of riddles for the ”Initial training” category with the variables used for each riddle, excluding variables contained in all riddles.

Topology

For each of the four riddles in the ”Topology” category listed in Supplementary Table 2, a point set is generated containing randomly selected points inside the frame. The point sets are used to create a polygon. Catmull-Rom spline is used for smoothing the polygons in all the riddles. The rotation variable is adopted in the *Connectedness* riddle to define a line, centered on the frame center, that separates the two shapes such that the two shapes are on either side of the line. Two point sets are generated for the *Holes* riddle, one for the inner shape and the second for the outer shape. Each riddle has a minimum area for a shape to ensure that the shapes are clearly visible.

**Riddle type**	**Point set**	**Point radius**	**Rotation**
Inside	X	X	
Closure	X		
Connectedness	X		X
Holes	X		

Supplementary Table 2: List of riddles for the ”Topology” category with the variables used for each riddle, excluding variables contained in all riddles.

Euclidean geometry

The ”Euclidean geometry” category includes eight riddles. However, only six are explicitly listed in Supplementary Table 3, as the *Right Angle* and *Alignment of point in line* riddles correspond to two versions that are split into separate riddles. Bezier curves were used to create curved lines. The offset variable refers to a point offset from the center. Each line has a length, width, center and rotation that are randomly generated. The lines in the secant lines have at least a 10 deg difference in rotation. *Parallel/secant lines* riddles use the offset variable as the center of the second line.

**Riddle type**	**Length**	**Width**	**Rot.**	**Offset**	**Center**	**P. radius**
Straight/curved	X	X	X		X	
Alignment of point on line	X	X	X	X	X	X
Parallel/secant	X	X	X	X	X	
Right angle	X	X	X		X	

Supplementary Table 3: List of riddles for the ”Euclidean geometry” category with the variables used for each riddle, excluding variables contained in all riddles. The Width variable refers to the width of the line. Rot. and P. refer to the Rotation and Point, respectively.

Geometrical figures

The ”Geometrical figures” category includes nine riddles listed in Supplementary Table 4. All polygons have a minimum area for visibility of the geometrical properties. Every line in a frame of the *Trapezoid* riddle is ensured not to be parallel to another line in the same frame, by defining a minimum slope difference between each line. *Parallelogram* riddle uses the angle variable to define an angle between the edges, whereas the *Right angled triangle* uses randomized angles within a circumcircle of the triangle as points. Height and width for the *Equilateral* riddle represent the height of the triangle and the bottom edge length. Height and width for the *Circle* riddle represent the dimensions of the minimum bounding rectangle of the ellipse. The *Convex shape* riddle is generated by calculating the convex hull of a random point set. For the oddity, a point in the convex hull is replaced by a point inside the area of the convex hull.

Riddle type	Point set	H & W	Angle	Radius	Center	Rot.
Quadrilateral	X					
Trapezoid		X	X		X	X
Parallelogram		X	X		X	X
Rectangle		X	X		X	X
Square		X			X	X
Equilateral triangle		X			X	X
Right-angled triangle			X	X	X	
Circle		X			X	X
Convex shape	X					

Supplementary Table 4: List of riddles for the ”Geometrical figures” category with the variables used for each riddle, excluding variables contained in all riddles. H & W and Rot. denote the Height and width and the Rotation variables, respectively.

Symmetrical figures

Each frame from the three riddles in the ”Symmetrical figures” category listed in Supplementary Table 5 has eight shapes, four on each side of a vertical line that is not shown in the frame. The four bottom shapes of the oddity do not correspond symmetrically to the four top shapes. Each of the eight shapes of the oddity has a randomized position on the line separating the top and bottom shapes. The top and bottom shapes of non-oddities are symmetrical. The height of each shape follows the same procedure as the shape positions. The height of a shape is the maximum distance between the shape and the line separating the top and bottom shapes. Rotation of 90 deg is used for the vertical riddle while in the oblique axis riddle the rotation must differ at least 10 deg from 0 deg + n×90 deg, $n \in Z$. This makes sure that the oblique riddle is never the same as the horizontal and vertical axis riddles.

Riddle type	Center	Rotation	Length	Height	Position
Symmetrical figures	X	X	X	X	X

Supplementary Table 5: List of riddles for the ”Symmetrical figures” category with the variables used for each riddle, excluding variables contained in all riddles. All three riddles in the category use the same variables. The Height and Position variables refer to height and position of the eight shapes.

Chiral figures

The four riddles in the ”Chiral figures” category can be split into two subcategories: circle and segments, each including two riddles, listed in Supplementary Table 6. The subcategories refer to the image on the side of the main segment. The images on the side are positioned by an offset from the center where the segment subcategory has two offsets. Each segment also has its own length variable randomized. The rotation variable is only used in the oblique axis riddles.

Riddle type	Center	Rot.	Radius	Length	Offset	Width
Chiral 1	X	X	X	X	X	X
Chiral 2	X	X		X	X	X

Supplementary Table 6: List of riddles for the ”Chiral figures” category with the variables used for each riddle, excluding variables contained in all riddles. The width refers to the width of the segments. Rot. refers to the Rotation variable.

Metric properties

The *Distance*, *Middle of segment* and *Fixed proportion* riddles in the ”Metric properties” category listed in Supplementary Table 7 adopt an offset from the center for each point. A minimum difference in the point offset values is enforced for better visibility between oddities and non-oddities. To generate the oddities of *Center of circle* and *Center of quadrilateral* riddles the center point of the non-oddities is offset by a minimum amount. Both the *Equidistance* and *Increasing distance* riddles are generated by modeling a line and using the points based on offsets from the center of the line.

Riddle type	P. set	Center	Rot.	Radius	Length	Offset	Width
Distance		X	X		X	X	X
Equidistance		X	X		X	X	X
Increasing distance		X	X		X	X	X
Circle center		X		X		X	
Center of quadrilateral	X				X	X	
Middle of segment		X	X		X	X	X
Fixed proportions		X			X	X	X

Supplementary Table 7: List of riddles for the ”Metric properties” category with the variables used for each riddle, excluding variables contained in all riddles. The modeled line width for the *Equidistance* and *Increasing distance* riddles is used as point radius. The width refers to the line segment width. P. set and Rot. refer to the Point set and Rotation variables, respectively.

Geometrical transformations

The ”Geometrical transformation” category includes eight riddles, which are listed in Supplementary Table 8. A minimum rotation is defined to facilitate the identification of the oddities of *Translation*, *Homothecy* and *Rotation*. The *Symmetry* riddles use a horizontal axis that is rotated randomly in the oblique riddle and 90 deg in the vertical riddle. All triangles are ensured to have a minimum area for visibility.

Riddle type	P. set	Rotation	Ratio	Length	P. radius
Translation	X	X			
Homothecy	X	X	X		
Symmetry	X	X		X	
Point symmetry	X	X			X
Rotation	X	X		X	X

Supplementary Table 8: List of riddles for the ”Geometrical transformations” category with the variables used for each riddle, excluding variables contained in all riddles. Symmetry refers to all three symmetry riddles: horizontal, vertical and oblique. Homothecy refers to both homothecy riddles shown in Supplementary Figure 1. P. set and P. radius refer to the Point set and Point radius variables, respectively.

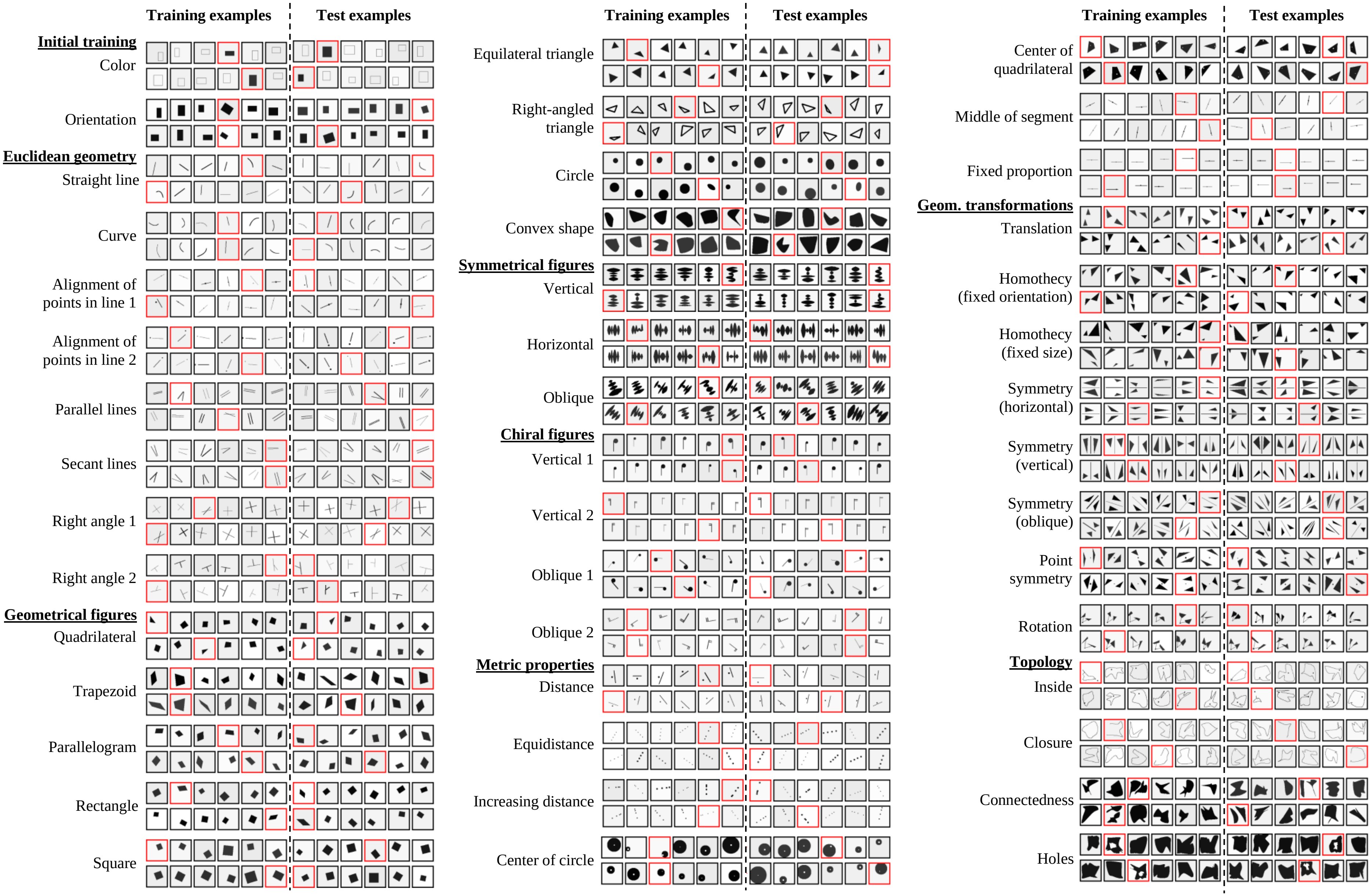

Supplementary Figure 1. Examples for each riddle, with riddles grouped by categories written in an underlined font. For each riddle, two training and two test examples composed of 6 horizontal panels are visualized. The red border highlights the oddity.

Supplementary Note 2: Detailed architecture of the vision model

Detailed architecture of the CNN vision model used by OReN and saccadic network is illustrated in Supplementary Figure 2.

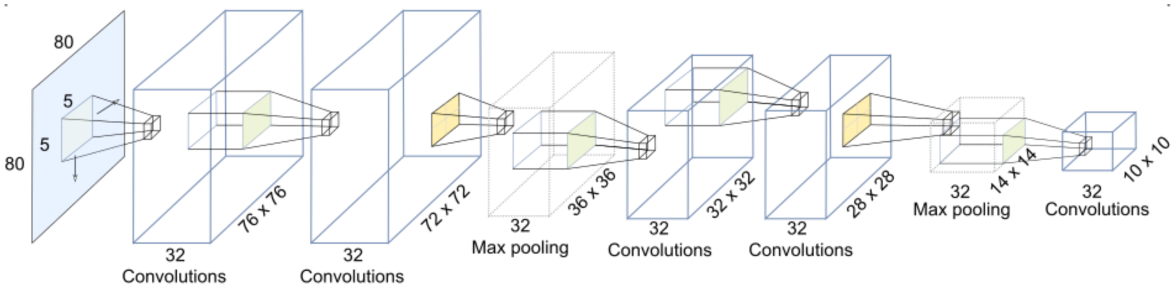

Supplementary Figure 2: Detailed architecture of the vision model.

Supplementary Note 3: Detailed results for the visual oddity task

Model	N	Parameters	Avg. accuracy	Seeds per riddle	1	2	3	4	5	6	7	8	9	10	11	12	13	14	15	16	17	18	19	20	21	22	23	24	25	26	27	28	29	30	31	32	33	34	35	36	37	38	39	40	41	42	43	44	45
OReN	16	207 313	95.4 ±15.8	10	100.0 ±0.0	100.0 ±0.0	99.9 ±0.1	100.0 ±0.1	100.0 ±0.1	74.9 ±37.9	99.7 ±0.3	99.9 ±0.1	99.9 ±0.1	99.8 ±0.2	100.0 ±0.1	99.6 ±0.4	90.0 ±4.3	100.0 ±0.1	99.7 ±0.3	99.8 ±0.4	98.1 ±1.1	99.8 ±0.2	97.8 ±0.5	99.9 ±0.1	99.8 ±0.2	99.4 ±0.4	100.0 ±0.0	100.0 ±0.0	91.8 ±24.6	99.9 ±0.1	99.8 ±0.1	99.9 ±0.1	94.6 ±0.9	91.3 ±25.4	84.8 ±22.3	99.8 ±0.1	41.8 ±37.8	92.3 ±3.2	78.0 ±10.0	98.4 ±0.9	99.8 ±0.1	99.8 ±0.2	98.2 ±0.7	99.4 ±0.5	67.8 ±35.3	99.8 ±0.3	99.5 ±0.2	99.7 ±0.2	99.8 ±0.2
OReN	32	313 665	97.2 ±10.6	10	100.0 ±0.0	99.9 ±0.2	99.8 ±0.1	99.8 ±0.2	100.0 ±0.1	99.8 ±0.1	99.6 ±0.2	99.7 ±0.3	99.9 ±0.1	99.8 ±0.2	100.0 ±0.0	99.6 ±0.2	88.7 ±4.5	99.9 ±0.2	99.6 ±0.4	100.0 ±0.1	98.2 ±0.5	99.7 ±0.3	98.0 ±0.5	99.9 ±0.2	99.8 ±0.2	99.0 ±1.6	100.0 ±0.1	100.0 ±0.0	99.9 ±0.1	99.9 ±0.1	99.8 ±0.1	99.8 ±0.2	94.2 ±1.0	99.9 ±0.1	84.9 ±22.7	99.9 ±0.1	58.7 ±41.1	93.1 ±1.8	82.0 ±2.5	98.7 ±0.6	99.8 ±0.2	99.8 ±0.2	98.1 ±0.7	99.5 ±0.4	88.3 ±23.3	99.7 ±0.2	99.5 ±0.3	99.7 ±0.2	100.0 ±0.1
OReN	64	534 049	97.4 ±10.0	10	100.0 ±0.0	99.9 ±0.1	99.8 ±0.2	99.8 ±0.2	100.0 ±0.0	99.9 ±0.2	99.6 ±0.2	99.9 ±0.1	100.0 ±0.1	99.9 ±0.1	99.9 ±0.1	99.7 ±0.2	89.6 ±1.3	99.8 ±0.2	99.7 ±0.2	99.8 ±0.4	98.0 ±1.0	99.7 ±0.2	97.5 ±0.8	99.9 ±0.1	99.9 ±0.2	99.5 ±0.4	100.0 ±0.0	99.9 ±0.1	100.0 ±0.1	99.8 ±0.1	99.9 ±0.2	100.0 ±0.1	94.7 ±0.7	99.8 ±0.1	87.1 ±3.7	99.8 ±0.2	50.0 ±40.2	94.2 ±2.4	83.7 ±1.7	98.8 ±0.5	99.8 ±0.2	99.9 ±0.1	98.5 ±0.5	99.5 ±0.5	96.5 ±0.9	99.7 ±0.2	99.3 ±0.3	99.6 ±0.3	99.9 ±0.2
OReN	128	1 005 537	98.3 ±5.3	10	100.0 ±0.0	99.9 ±0.1	99.9 ±0.1	99.8 ±0.1	100.0 ±0.1	99.8 ±0.2	99.5 ±0.4	99.7 ±0.3	99.9 ±0.1	99.8 ±0.1	100.0 ±0.1	99.6 ±0.2	88.7 ±3.9	99.8 ±0.2	99.6 ±0.2	99.8 ±0.3	97.6 ±1.1	99.6 ±0.3	97.7 ±0.5	99.7 ±0.2	99.8 ±0.4	99.1 ±0.7	100.0 ±0.1	100.0 ±0.1	99.9 ±0.1	99.8 ±0.4	99.8 ±0.2	99.9 ±0.2	93.8 ±1.3	99.8 ±0.2	89.0 ±2.1	99.9 ±0.1	91.2 ±25.7	95.1 ±1.3	83.5 ±2.5	99.0 ±0.5	99.9 ±0.2	99.7 ±0.3	98.4 ±0.4	99.3 ±0.3	96.2 ±0.5	99.8 ±0.2	99.4 ±0.3	99.6 ±0.2	99.9 ±0.1
OReN	256	2 071 393	98.3 ±5.3	10	100.0 ±0.0	100.0 ±0.0	99.8 ±0.2	99.9 ±0.1	99.9 ±0.1	99.8 ±0.2	99.6 ±0.2	99.8 ±0.1	99.9 ±0.2	99.9 ±0.1	100.0 ±0.1	99.6 ±0.2	89.1 ±2.0	99.9 ±0.2	99.8 ±0.2	99.9 ±0.1	96.9 ±0.9	99.7 ±0.3	97.7 ±0.6	99.8 ±0.3	99.9 ±0.1	99.1 ±0.6	100.0 ±0.0	99.9 ±0.2	99.9 ±0.1	99.9 ±0.1	99.8 ±0.2	99.8 ±0.3	93.9 ±1.0	99.8 ±0.2	88.8 ±2.3	99.9 ±0.1	91.5 ±25.0	95.0 ±1.8	82.4 ±3.3	98.9 ±0.6	99.8 ±0.2	99.8 ±0.3	98.2 ±0.6	99.3 ±0.4	96.1 ±0.9	99.8 ±0.3	99.4 ±0.3	99.6 ±0.3	100.0 ±0.1
LSTM	16	314 129	98.0 ±8.2	10	100.0 ±0.0	100.0 ±0.0	100.0 ±0.1	99.9 ±0.4	100.0 ±0.0	99.9 ±0.1	99.8 ±0.2	99.9 ±0.1	100.0 ±0.0	99.9 ±0.1	99.9 ±0.1	99.8 ±0.2	92.7 ±2.5	100.0 ±0.0	99.6 ±0.4	99.9 ±0.1	99.6 ±0.3	99.9 ±0.1	98.5 ±0.4	99.9 ±0.1	100.0 ±0.0	99.7 ±0.2	98.9 ±3.2	99.9 ±0.1	100.0 ±0.0	99.9 ±0.2	99.9 ±0.1	100.0 ±0.0	95.3 ±1.0	99.9 ±0.1	91.8 ±3.9	100.0 ±0.1	64.7 ±38.1	92.4 ±1.6	84.6 ±3.2	99.5 ±0.3	99.9 ±0.1	99.9 ±0.1	99.1 ±0.7	99.7 ±0.3	97.1 ±0.6	99.8 ±0.2	99.8 ±0.2	99.8 ±0.2	99.9 ±0.2
LSTM	32	534 977	97.9 ±8.6	10	100.0 ±0.0	99.9 ±0.3	99.9 ±0.1	99.9 ±0.1	100.0 ±0.0	99.9 ±0.1	99.8 ±0.1	99.8 ±0.2	100.0 ±0.0	99.9 ±0.2	99.9 ±0.1	99.9 ±0.2	92.3 ±1.9	100.0 ±0.0	99.7 ±0.3	100.0 ±0.0	99.3 ±0.4	99.9 ±0.1	98.4 ±0.5	99.9 ±0.1	99.9 ±0.1	99.4 ±0.5	99.2 ±1.8	100.0 ±0.0	100.0 ±0.0	100.0 ±0.1	100.0 ±0.1	100.0 ±0.0	95.2 ±0.8	99.9 ±0.1	92.0 ±3.2	100.0 ±0.1	65.9 ±40.7	92.9 ±0.8	80.4 ±10.4	99.7 ±0.3	99.9 ±0.1	99.9 ±0.1	99.1 ±0.3	99.4 ±0.3	96.7 ±0.5	99.9 ±0.1	99.7 ±0.2	99.6 ±0.2	100.0 ±0.1
LSTM	64	1 007 393	98.0 ±7.5	10	100.0 ±0.0	100.0 ±0.1	99.9 ±0.1	99.8 ±0.2	100.0 ±0.0	99.9 ±0.1	99.7 ±0.2	99.8 ±0.2	100.0 ±0.1	99.9 ±0.1	100.0 ±0.0	99.8 ±0.3	90.5 ±1.1	99.9 ±0.1	99.7 ±0.2	100.0 ±0.0	98.9 ±0.8	99.8 ±0.3	98.6 ±0.4	99.9 ±0.3	100.0 ±0.1	99.3 ±0.6	99.8 ±0.5	99.9 ±0.2	99.9 ±0.2	100.0 ±0.1	99.9 ±0.1	99.9 ±0.1	94.9 ±1.0	99.9 ±0.1	90.0 ±3.9	100.0 ±0.1	78.7 ±32.3	92.8 ±1.3	77.0 ±19.5	99.8 ±0.1	99.9 ±0.2	99.9 ±0.1	98.8 ±0.5	99.6 ±0.3	96.8 ±0.9	99.8 ±0.2	99.7 ±0.2	99.8 ±0.3	100.0 ±0.0
LSTM	128	2 075 105	98.0 ±7.9	10	100.0 ±0.0	100.0 ±0.0	99.9 ±0.1	99.9 ±0.2	100.0 ±0.0	99.9 ±0.2	99.7 ±0.3	99.9 ±0.1	99.9 ±0.1	99.9 ±0.1	99.9 ±0.1	99.9 ±0.1	88.3 ±1.3	99.9 ±0.2	99.7 ±0.3	99.9 ±0.1	99.0 ±0.5	99.8 ±0.3	98.3 ±0.4	99.9 ±0.1	100.0 ±0.1	99.6 ±0.5	99.9 ±0.3	100.0 ±0.0	100.0 ±0.0	99.9 ±0.1	100.0 ±0.1	99.9 ±0.1	94.9 ±0.9	99.9 ±0.1	91.5 ±4.0	100.0 ±0.1	74.8 ±37.9	92.8 ±1.0	78.0 ±8.6	99.7 ±0.2	99.9 ±0.1	100.0 ±0.1	98.8 ±0.5	99.6 ±0.3	96.5 ±0.7	99.8 ±0.2	99.8 ±0.2	99.8 ±0.2	100.0 ±0.0
LSTM	256	4 702 049	98.2 ±6.4	10	100.0 ±0.0	100.0 ±0.0	100.0 ±0.1	99.9 ±0.2	100.0 ±0.0	100.0 ±0.1	99.8 ±0.1	100.0 ±0.0	100.0 ±0.0	99.9 ±0.1	100.0 ±0.1	99.8 ±0.2	88.5 ±1.2	100.0 ±0.0	99.6 ±0.3	99.9 ±0.1	98.6 ±0.9	99.9 ±0.2	98.2 ±0.5	100.0 ±0.1	100.0 ±0.0	99.7 ±0.2	99.6 ±1.3	100.0 ±0.0	99.9 ±0.3	99.9 ±0.1	100.0 ±0.1	100.0 ±0.1	94.2 ±1.2	99.9 ±0.1	88.5 ±4.1	100.0 ±0.1	84.1 ±31.6	92.3 ±2.5	81.8 ±1.7	99.7 ±0.2	99.9 ±0.1	99.9 ±0.1	98.4 ±0.8	99.5 ±0.3	97.1 ±0.6	99.9 ±0.1	99.5 ±0.3	99.8 ±0.2	100.0 ±0.0
SNN	16	155 489	92.9 ±16.6	10	100.0 ±0.0	100.0 ±0.0	99.9 ±0.1	99.9 ±0.2	100.0 ±0.1	99.2 ±1.7	99.5 ±0.3	99.8 ±0.1	99.9 ±0.1	99.9 ±0.1	99.9 ±0.0	99.7 ±0.3	64.5 ±7.3	99.9 ±0.1	94.5 ±5.5	99.9 ±0.1	92.9 ±8.4	97.5 ±4.0	97.7 ±0.7	99.5 ±0.4	91.5 ±25.1	87.5 ±10.6	100.0 ±0.0	100.0 ±0.1	100.0 ±0.0	100.0 ±0.1	96.4 ±2.2	100.0 ±0.1	93.3 ±1.3	99.8 ±0.2	70.8 ±18.6	99.4 ±0.6	17.2 ±1.4	70.8 ±6.4	53.6 ±8.8	96.4 ±0.9	99.9 ±0.1	99.8 ±0.2	98.4 ±0.7	88.4 ±4.0	74.8 ±11.9	99.7 ±0.2	99.3 ±0.4	99.4 ±0.5	99.8 ±0.2
SNN	32	208 481	95.7 ±10.9	10	100.0 ±0.0	99.9 ±0.3	99.9 ±0.2	99.9 ±0.1	100.0 ±0.0	99.9 ±0.1	99.7 ±0.2	99.7 ±0.3	100.0 ±0.0	100.0 ±0.0	99.9 ±0.1	99.6 ±0.3	70.0 ±11.5	99.9 ±0.1	99.1 ±1.5	99.8 ±0.1	95.0 ±6.7	98.8 ±1.5	98.3 ±0.6	99.9 ±0.2	99.0 ±2.1	88.7 ±7.6	100.0 ±0.0	100.0 ±0.0	100.0 ±0.1	100.0 ±0.1	97.6 ±3.0	100.0 ±0.0	93.9 ±1.1	99.9 ±0.1	84.7 ±7.7	99.9 ±0.1	59.9 ±34.8	80.0 ±3.9	69.8 ±5.7	97.5 ±0.4	99.9 ±0.1	99.8 ±0.2	98.8 ±0.6	94.3 ±1.6	85.2 ±6.4	99.9 ±0.1	99.5 ±0.3	99.5 ±0.3	99.8 ±0.2
SNN	64	317 537	98.1 ±6.3	10	100.0 ±0.1	100.0 ±0.0	99.9 ±0.1	100.0 ±0.1	100.0 ±0.0	99.9 ±0.2	99.8 ±0.1	99.9 ±0.2	100.0 ±0.0	99.9 ±0.2	99.8 ±0.1	99.7 ±0.3	89.2 ±10.3	100.0 ±0.0	99.9 ±0.1	100.0 ±0.1	99.8 ±0.2	99.9 ±0.2	98.7 ±0.2	99.9 ±0.1	100.0 ±0.1	98.0 ±4.2	100.0 ±0.0	100.0 ±0.0	100.0 ±0.0	99.9 ±0.1	99.7 ±0.3	99.9 ±0.1	95.4 ±0.8	99.8 ±0.2	96.4 ±2.0	99.8 ±0.2	82.9 ±27.5	87.6 ±1.6	79.8 ±2.1	98.6 ±0.7	99.8 ±0.2	99.8 ±0.2	99.1 ±0.3	99.0 ±0.6	96.0 ±1.5	99.8 ±0.3	99.7 ±0.2	99.5 ±0.3	100.0 ±0.1
SNN	128	547 937	98.9 ±3.1	10	99.9 ±0.1	100.0 ±0.0	99.9 ±0.1	99.9 ±0.1	100.0 ±0.0	99.9 ±0.1	99.8 ±0.1	99.9 ±0.1	100.0 ±0.1	99.7 ±0.3	99.9 ±0.2	99.8 ±0.3	93.7 ±1.3	99.9 ±0.1	99.9 ±0.1	100.0 ±0.1	99.8 ±0.2	99.9 ±0.1	98.4 ±0.4	99.9 ±0.1	100.0 ±0.1	99.6 ±0.2	100.0 ±0.0	100.0 ±0.0	100.0 ±0.1	99.9 ±0.1	99.8 ±0.2	100.0 ±0.0	95.5 ±1.0	99.9 ±0.2	98.5 ±0.6	100.0 ±0.1	98.9 ±2.1	91.3 ±2.1	82.5 ±2.6	99.4 ±0.3	99.8 ±0.2	99.9 ±0.1	99.2 ±0.2	99.5 ±0.2	97.5 ±0.6	99.8 ±0.1	99.8 ±0.2	99.7 ±0.2	100.0 ±0.1
SNN	256	1 057 889	98.7 ±5.2	10	100.0 ±0.0	100.0 ±0.1	100.0 ±0.1	99.9 ±0.1	100.0 ±0.1	99.9 ±0.1	99.8 ±0.2	99.8 ±0.2	100.0 ±0.1	100.0 ±0.1	99.9 ±0.1	99.8 ±0.2	95.0 ±1.9	100.0 ±0.0	99.8 ±0.1	100.0 ±0.1	99.7 ±0.3	99.9 ±0.1	98.6 ±0.6	99.9 ±0.2	100.0 ±0.1	99.7 ±0.2	100.0 ±0.0	100.0 ±0.0	100.0 ±0.0	100.0 ±0.0	100.0 ±0.1	100.0 ±0.0	95.2 ±0.7	99.9 ±0.1	98.8 ±0.6	100.0 ±0.1	91.4 ±25.1	90.7 ±1.4	79.5 ±4.1	99.4 ±0.4	99.9 ±0.2	99.9 ±0.2	99.2 ±0.5	99.5 ±0.4	97.7 ±0.5	99.9 ±0.1	99.7 ±0.3	99.8 ±0.1	99.9 ±0.2
sSNU	16	155 489	98.7 ±5.0	10	100.0 ±0.0	100.0 ±0.1	99.9 ±0.1	100.0 ±0.0	100.0 ±0.1	100.0 ±0.0	99.9 ±0.2	99.8 ±0.2	100.0 ±0.1	99.9 ±0.1	99.8 ±0.2	99.8 ±0.3	96.0 ±1.5	100.0 ±0.1	99.9 ±0.1	99.9 ±0.2	99.8 ±0.3	99.8 ±0.2	98.8 ±0.5	99.9 ±0.1	99.9 ±0.1	99.8 ±0.2	100.0 ±0.0	100.0 ±0.0	99.9 ±0.1	100.0 ±0.0	99.1 ±0.3	99.9 ±0.1	95.5 ±0.7	100.0 ±0.1	90.1 ±24.5	99.9 ±0.2	99.3 ±0.4	91.6 ±1.6	81.2 ±2.6	98.2 ±0.9	99.8 ±0.3	99.8 ±0.2	99.3 ±0.5	99.4 ±0.3	97.3 ±1.5	99.9 ±0.1	99.7 ±0.2	99.7 ±0.1	100.0 ±0.1
sSNU	32	208 481	99.0 ±3.0	10	100.0 ±0.0	100.0 ±0.0	100.0 ±0.0	100.0 ±0.1	100.0 ±0.0	100.0 ±0.0	99.8 ±0.2	99.9 ±0.2	100.0 ±0.0	100.0 ±0.0	99.9 ±0.1	99.8 ±0.2	95.2 ±2.1	100.0 ±0.0	99.9 ±0.1	100.0 ±0.1	99.8 ±0.2	100.0 ±0.1	98.7 ±0.4	99.9 ±0.1	99.9 ±0.1	99.7 ±0.2	100.0 ±0.0	100.0 ±0.0	100.0 ±0.0	100.0 ±0.1	99.6 ±0.4	100.0 ±0.1	95.4 ±0.7	99.9 ±0.1	98.5 ±0.9	99.9 ±0.1	99.7 ±0.3	92.2 ±1.6	82.5 ±2.4	99.2 ±0.4	99.9 ±0.2	99.9 ±0.1	99.3 ±0.5	99.5 ±0.2	97.5 ±0.6	99.9 ±0.2	99.8 ±0.1	99.7 ±0.2	99.9 ±0.1
sSNU	64	317 537	99.0 ±3.1	10	100.0 ±0.0	100.0 ±0.0	100.0 ±0.1	100.0 ±0.1	100.0 ±0.0	100.0 ±0.1	99.8 ±0.2	99.9 ±0.2	100.0 ±0.0	99.9 ±0.2	99.9 ±0.2	99.9 ±0.2	96.2 ±1.2	100.0 ±0.1	99.7 ±0.3	100.0 ±0.0	99.8 ±0.2	99.9 ±0.1	98.8 ±0.3	99.8 ±0.2	99.9 ±0.1	99.7 ±0.3	100.0 ±0.1	99.9 ±0.2	100.0 ±0.0	100.0 ±0.0	99.8 ±0.2	99.9 ±0.1	95.6 ±0.9	100.0 ±0.1	98.4 ±0.8	100.0 ±0.1	99.7 ±0.2	91.6 ±2.4	81.7 ±3.2	99.5 ±0.4	99.9 ±0.2	100.0 ±0.1	99.3 ±0.4	99.5 ±0.2	97.6 ±0.9	99.9 ±0.1	99.8 ±0.2	99.8 ±0.2	100.0 ±0.1
sSNU	128	547 937	98.8 ±4.2	10	100.0 ±0.0	100.0 ±0.0	100.0 ±0.1	100.0 ±0.1	100.0 ±0.0	99.9 ±0.2	99.9 ±0.2	99.9 ±0.1	100.0 ±0.1	99.9 ±0.1	99.8 ±0.1	99.8 ±0.2	93.4 ±2.0	99.9 ±0.1	99.9 ±0.2	100.0 ±0.1	99.7 ±0.3	99.9 ±0.1	98.6 ±0.4	99.9 ±0.1	100.0 ±0.1	99.7 ±0.3	100.0 ±0.0	100.0 ±0.0	100.0 ±0.0	100.0 ±0.0	99.8 ±0.2	100.0 ±0.0	95.6 ±0.7	99.9 ±0.1	97.8 ±0.9	100.0 ±0.0	99.8 ±0.2	91.3 ±1.7	77.0 ±13.7	99.4 ±0.4	100.0 ±0.1	99.9 ±0.1	99.4 ±0.4	99.5 ±0.3	98.1 ±0.6	99.9 ±0.2	99.9 ±0.1	99.7 ±0.3	100.0 ±0.1
sSNU	256	1 057 889	98.7 ±5.0	10	100.0 ±0.0	100.0 ±0.0	99.9 ±0.2	100.0 ±0.1	100.0 ±0.0	100.0 ±0.0	99.9 ±0.1	100.0 ±0.0	100.0 ±0.1	99.9 ±0.1	99.9 ±0.1	99.9 ±0.1	93.2 ±1.2	100.0 ±0.0	99.9 ±0.1	100.0 ±0.0	99.7 ±0.2	99.9 ±0.2	98.5 ±0.6	99.9 ±0.2	99.9 ±0.1	99.9 ±0.2	100.0 ±0.0	100.0 ±0.0	100.0 ±0.1	100.0 ±0.1	99.8 ±0.3	100.0 ±0.0	95.4 ±0.9	99.9 ±0.1	97.4 ±2.1	100.0 ±0.1	99.8 ±0.1	93.1 ±1.7	72.4 ±17.2	99.5 ±0.5	99.9 ±0.1	99.9 ±0.1	99.5 ±0.4	99.6 ±0.2	98.0 ±0.7	99.9 ±0.1	99.8 ±0.3	99.6 ±0.3	100.0 ±0.1
sSNU-R	16	156 257	98.7 ±4.9	10	100.0 ±0.0	100.0 ±0.0	100.0 ±0.0	99.9 ±0.1	100.0 ±0.0	100.0 ±0.1	99.8 ±0.2	99.9 ±0.2	100.0 ±0.1	100.0 ±0.1	100.0 ±0.1	99.9 ±0.1	95.4 ±2.0	99.9 ±0.2	99.8 ±0.3	100.0 ±0.1	99.8 ±0.4	99.8 ±0.3	98.8 ±0.3	99.9 ±0.2	99.9 ±0.1	99.7 ±0.3	100.0 ±0.0	100.0 ±0.1	100.0 ±0.1	99.9 ±0.1	99.7 ±0.3	100.0 ±0.1	95.5 ±0.8	99.9 ±0.1	97.8 ±1.1	99.9 ±0.2	91.2 ±24.6	90.7 ±1.6	81.9 ±2.0	98.9 ±0.5	100.0 ±0.1	99.9 ±0.2	99.3 ±0.5	99.6 ±0.3	97.3 ±0.7	100.0 ±0.1	99.9 ±0.1	99.8 ±0.2	100.0 ±0.0
sSNU-R	32	211 553	99.0 ±2.9	10	100.0 ±0.0	100.0 ±0.0	100.0 ±0.1	99.9 ±0.2	100.0 ±0.0	99.9 ±0.1	99.9 ±0.2	99.8 ±0.2	100.0 ±0.1	99.9 ±0.2	99.9 ±0.1	99.9 ±0.2	94.7 ±1.5	100.0 ±0.1	99.6 ±0.4	100.0 ±0.0	99.7 ±0.2	99.9 ±0.1	98.7 ±0.4	99.9 ±0.2	99.9 ±0.1	99.6 ±0.3	100.0 ±0.0	100.0 ±0.1	100.0 ±0.1	100.0 ±0.0	99.8 ±0.3	99.9 ±0.2	95.6 ±0.6	99.9 ±0.1	98.2 ±1.3	99.9 ±0.1	99.7 ±0.1	92.7 ±1.5	82.6 ±1.9	99.4 ±0.4	99.9 ±0.1	99.9 ±0.1	99.5 ±0.3	99.6 ±0.4	97.6 ±0.5	99.8 ±0.1	99.8 ±0.2	99.8 ±0.4	100.0 ±0.0
sSNU-R	64	329 825	98.6 ±6.3	10	100.0 ±0.0	100.0 ±0.1	99.9 ±0.1	99.9 ±0.2	100.0 ±0.0	99.9 ±0.1	99.8 ±0.2	99.8 ±0.2	100.0 ±0.0	99.9 ±0.1	99.9 ±0.1	99.9 ±0.2	93.8 ±1.8	100.0 ±0.1	99.8 ±0.3	100.0 ±0.1	99.8 ±0.1	99.9 ±0.2	98.6 ±0.4	100.0 ±0.1	99.9 ±0.1	99.8 ±0.3	100.0 ±0.0	100.0 ±0.0	100.0 ±0.0	100.0 ±0.0	99.9 ±0.1	99.9 ±0.1	95.5 ±0.8	99.9 ±0.2	98.3 ±1.0	99.9 ±0.2	83.2 ±33.3	92.6 ±1.6	81.8 ±2.8	99.7 ±0.3	99.9 ±0.1	99.9 ±0.1	99.4 ±0.4	99.5 ±0.3	97.6 ±0.4	99.9 ±0.2	99.9 ±0.1	99.8 ±0.4	100.0 ±0.0
sSNU-R	128	597 089	98.7 ±5.7	10	100.0 ±0.0	100.0 ±0.0	100.0 ±0.1	99.9 ±0.1	100.0 ±0.0	99.9 ±0.1	99.8 ±0.2	100.0 ±0.1	100.0 ±0.0	99.9 ±0.1	99.9 ±0.2	99.9 ±0.1	93.5 ±3.4	100.0 ±0.1	99.9 ±0.1	100.0 ±0.1	99.7 ±0.2	100.0 ±0.0	98.6 ±0.6	99.9 ±0.1	100.0 ±0.1	99.8 ±0.3	100.0 ±0.1	100.0 ±0.1	100.0 ±0.0	99.9 ±0.1	99.9 ±0.2	99.9 ±0.1	95.8 ±0.9	99.9 ±0.1	96.4 ±3.4	99.9 ±0.1	93.6 ±18.8	92.8 ±1.4	74.9 ±18.5	99.7 ±0.2	99.9 ±0.1	99.9 ±0.1	99.4 ±0.3	99.7 ±0.2	97.7 ±0.8	99.8 ±0.2	99.9 ±0.1	99.7 ±0.2	100.0 ±0.0
sSNU-R	256	1 254 497	98.2 ±8.0	10	100.0 ±0.0	99.9 ±0.1	99.9 ±0.1	99.9 ±0.1	100.0 ±0.0	100.0 ±0.1	99.7 ±0.2	99.9 ±0.1	100.0 ±0.1	99.9 ±0.1	100.0 ±0.1	99.9 ±0.1	92.1 ±1.8	100.0 ±0.1	99.9 ±0.2	99.9 ±0.1	99.8 ±0.3	99.9 ±0.2	98.5 ±0.4	100.0 ±0.1	100.0 ±0.0	99.8 ±0.2	100.0 ±0.0	100.0 ±0.1	100.0 ±0.0	100.0 ±0.0	100.0 ±0.1	100.0 ±0.0	95.7 ±0.9	99.9 ±0.1	84.4 ±24.9	99.9 ±0.2	91.2 ±25.8	92.3 ±2.6	69.1 ±17.3	99.8 ±0.2	99.9 ±0.1	99.9 ±0.1	99.3 ±0.5	99.6 ±0.2	97.6 ±0.6	99.8 ±0.2	99.8 ±0.2	99.8 ±0.2	100.0 ±0.1

Supplementary Table 9: Separate training results. Test accuracy [%] ± standard deviation [%] reported at the best validation accuracy for models trained on riddles 1-45. The models were trained for 100 epochs with learning rate set to 0.001, batch size 16 and an exponential learning rate decay with base 0.97 applied after every 500 optimization steps.

Model	N	Parameters	Avg. accuracy	Seeds per riddle	Min	Max	Std
OReN	64	534 049	94.09%	10	92.75%	94.91%	0.58%
OReN	128	1 005 537	94.64%	10	94.07%	95.34%	0.42%
OReN	256	2 071 393	94.99%	10	94.73%	95.22%	0.13%
OReN	512	4 694 625	95.11%	10	94.37%	95.66%	0.43%
OReN	1024	11 907 169	95.15%	10	94.79%	95.46%	0.20%
OReN	2048	34 196 577	95.03%	10	94.62%	95.31%	0.20%
OReN	4096	110 232 673	94.41%	10	94.04%	94.88%	0.27%
LSTM	64	1 007 393	95.86%	10	95.19%	96.16%	0.28%
LSTM	128	2 075 105	96.06%	10	95.57%	96.52%	0.23%
LSTM	241	4 602 049	96.32%	10	96.04%	96.72%	0.20%
LSTM	256	4 702 049	96.29%	10	95.69%	97.07%	0.34%
LSTM	512	11 922 017	96.41%	10	96.08%	96.61%	0.16%
LSTM	1024	34 226 273	96.34%	10	96.06%	96.71%	0.22%
LSTM	2048	110 292 065	96.35%	10	95.81%	96.72%	0.25%
SNN	64	317 537	75.96%	10	71.07%	83.93%	4.13%
SNN	128	547 937	86.54%	10	85.77%	87.32%	0.60%
SNN	256	1 057 889	91.48%	10	90.63%	91.99%	0.41%
SNN	512	2 274 401	94.35%	10	93.92%	94.90%	0.28%
SNN	1024	5 493 857	95.30%	10	94.97%	95.72%	0.23%
SNN	2048	15 078 497	95.00%	10	94.78%	95.22%	0.15%
SNN	4096	46 830 689	94.36%	10	93.56%	94.76%	0.35%
sSNU	64	317 537	96.07%	10	95.71%	96.54%	0.25%
sSNU	128	547 937	96.04%	10	95.72%	96.31%	0.21%
sSNU	256	1 057 889	96.30%	10	95.93%	96.74%	0.31%
sSNU	512	2 274 401	96.26%	10	95.76%	96.62%	0.29%
sSNU	1024	5 493 857	96.29%	10	95.74%	96.85%	0.33%
sSNU	2048	15 078 497	96.23%	10	95.97%	96.51%	0.18%
sSNU	4096	46 830 689	95.81%	10	95.53%	96.16%	0.18%
sSNU-R	64	329 825	96.42%	10	96.04%	96.64%	0.18%
sSNU-R	128	597 089	96.52%	10	96.08%	96.97%	0.22%
sSNU-R	256	1 254 497	96.75%	10	96.03%	97.18%	0.33%
sSNU-R	512	3 060 833	96.95%	10	96.45%	97.35%	0.26%
sSNU-R	1024	8 639 585	96.98%	10	96.51%	97.34%	0.22%
sSNU-R	2048	27 661 409	97.00%	10	96.68%	97.42%	0.20%
sSNU-R	4096	97 162 337	97.00%	10	96.91%	97.16%	0.08%

Supplementary Table 10: Joint training results. Test accuracy reported at the best validation accuracy for models trained simultaneously on all 45 riddles. The models were trained for 100 epochs with learning rate set to 0.0001 and no learning rate decay.
